# Hospital costs associated with psychiatric comorbidities: a retrospective study

**DOI:** 10.1186/s12913-018-2892-5

**Published:** 2018-01-30

**Authors:** Jan Wolff, Thomas Heister, Claus Normann, Klaus Kaier

**Affiliations:** 1 Department of Psychiatry and Psychotherapy, Medical Center – University of Freiburg, Faculty of Medicine, University of Freiburg, Freiburg im Breisgau, Germany; 2 Department of Controlling, Evangelical Foundation Neuerkerode, Braunschweig, Germany; 3 Institute for Medical Biometry and Statistics, Medical Center – University of Freiburg, Faculty of Medicine, University of Freiburg, Freiburg im Breisgau, Germany

**Keywords:** Hospital costs, Comorbidity, Diagnosis-related groups, Psychiatry

## Abstract

**Background:**

Psychiatric comorbidities are relevant for the diagnostic and therapeutic regimes in somatic hospital care. The main aim of this study was to analyse the association between psychiatric comorbidities and hospital costs per inpatient episode. A further aim was to discuss and address the methodological challenges in the estimation of these outcomes based on retrospective data.

**Methods:**

The study included 338,162 inpatient episodes consecutively discharged between 2011 and 2014 at a German university hospital. We used detailed resource use data to calculate day-specific hospital costs. We adjusted analyses for sex, age, somatic comorbidities and main diagnoses. We addressed potential time-related bias in retrospective diagnosis data with sensitivity analyses.

**Results:**

Psychiatric comorbidities were associated with an increase in hospital costs per episode of 40% and an increase of reimbursement per episode of 28%, representing marginal effects of 1344 € and 1004 €, respectively. After controlling for length of stay, sensitivity analyses provided a lower bound increase in daily costs and reimbursement of 207 € and 151 €, respectively.

**Conclusion:**

If differences in hospital costs between patient groups are not adequately accounted for in DRG-systems, perverse incentives are created that can reduce the efficiency of care. Therefore, we suggest intensifying the inclusion of psychiatric comorbidities in the German DRG system. Future research should investigate the appropriate inclusion of psychiatric comorbidities in other health care systems’ payment schemes.

## Background

Mental and substance use disorders were the fifth leading cause of global disease burden and the leading cause of non-fatal burden in 2010 [1]. The cumulated global reduction of economic output due to mental disorders was estimated to be US $16 trillion from 2010 to 2030 [2]. Despite these high social costs, mental health has not achieved commensurate visibility, policy attention or funding [3]. In probably no country worldwide is the financial allocation for mental health proportionate to the contribution of mental disorders to the burden of disease [4].

Hospital care absorbs substantial shares of total health care budgets [5]. Diagnosis-related Groups (DRGs) are a dominant system for hospital reimbursement internationally [6]. DRGs use patient classifications systems with the aim to create cost homogenous groups that serve to define lump-sum hospital reimbursement per group [7]. Inadequate hospital reimbursement can result in inefficient care. Service providers may reduce costs at the expense of quality, cut length of stay for financial reasons and avoid treatment of high-cost patients [8–10]. If differences in hospital costs between patients are not adequately controlled for in DRG-systems, i.e. the payment is either too high or too low for many patients, such perverse incentives are created that can result in inefficient delivery of hospital services [11].

The prevalence of psychiatric comorbidities in somatic hospital care has been found to be between 15 and 50% [12–21]. Graham et al. [22] found that patients with mental health problems that co-occurred with somatic primary diseases were more than twice as likely to be admitted to somatic hospital care than patients without mental health problems.. An efficient provision of hospital care for patients with psychiatric comorbidities requires adequate consideration of respective differences in hospital costs.

Length of stay is a straightforward and frequently used measure of hospital resource use. Saravay et al. [23] reviewed 26 studies that investigated the effects of psychiatric comorbidity on length of stay in somatic hospital care and concluded that the majority of studies found a significant association between psychiatric comorbidity and increased length of stay. Koopmans et al. [24] reviewed 15 studies and found that psychiatric comorbidities were related to increased length of stay in some studies, but other studies did not find such a relation. Yet length of stay is not the same as total cost per episode, since per diem costs might also show substantial variations between hospital episodes. A length of stay analysis as a proxy for costs per episode would then underestimate the real cost differences.

Several studies have analysed the association between psychiatric comorbidities and financial costs of somatic hospital care [25–30]. They mainly found that psychiatric comorbidities were associated with increased costs. However, the identified studies used hospital charges or fixed per diem rates instead of costs, or they focused on patients with specific primary somatic diseases or psychiatric comorbidities.

Many studies on this topic use data routinely collected at hospitals or from insurance claims [31–39]. Secondary diagnoses, which are all diagnoses other than the principal reason for hospitalization, are then used for the analysis of comorbidities. Retrospective observational studies have the advantage of being an inexpensive way of collecting data and enabling large numbers of observations. However, there are several issues that can bias analyses.

First, observational data are generally prone to confounding, so it is important to disentangle the effect of the variable analysed from other effects. It is for example possible that older patients have more psychiatric comorbidities and at the same time higher costs of care for somatic reasons. Not controlling for age would then bias results.

Second, the analysis of routine data might be biased due to temporal aspects of detection and reporting of the condition. Since psychopathology is usually not systematically detected in routine clinical care, studies based on retrospective observational data rely on doctors spontaneously observing, detecting and documenting psychiatric comorbidities [24]. The possibility of detecting an existing psychiatric comorbidity might be seen as a function of length of stay. This would mean that patients staying longer would have a higher probability of detection.

On the other hand, there are psychiatric comorbidities which can occur as a complication of a procedure or a specific disease, notably delirium after a surgery or other intensive care treatment [40]. Including this as comorbidity in an analysis would then measure the incremental effect of procedural complication rather than that of psychiatric comorbidities [41]. Both these aspects of the time-dependency of psychiatric secondary diagnoses would bias results towards higher incremental costs compared with patients without psychiatric comorbidities.

The main aim of this study was to analyse the association between psychiatric comorbidities and hospital costs and revenues per inpatient episode. A further aim was to discuss and address the methodological challenges in the estimation of these outcomes based on retrospective data.

## Methods

### Data

The study site was a German tertiary-care university hospital that provides services for 70,000 inpatient hospital episodes and 800,000 outpatients per year. The dataset included all 338,162 inpatient episodes consecutively discharged between 2011 and 2014 at the study site. We excluded 47,335 episodes because they were younger than 18 years old and 8744 episodes because they had a psychiatric comorbidity possibly caused by the hospital stay. We excluded 37,504 episodes, because they had a main diagnosis in which no episode allowed statistical comparison in case-control analyses. Moreover, 84,025 episodes were treated in the psychiatric department of the university hospital and we excluded them because they were not reimbursed with DRGs. Furthermore, we excluded 1852 episodes because they had missing or implausible data (0.5%).

Implementation of the study required (1) data describing the characteristics of hospital episodes and (2) episode-specific resource use data. We obtained patient characteristics from the data-warehouse of the hospital. These data included all main and secondary diagnoses, age, sex, length of stay, intensive care hours and discharge status. We defined the presence of psychiatric comorbidities as the presence of at least one code from Chapter V (F0-F9) of the International Statistical Classification Of Diseases And Related Health Problems, 10th revision, German Modification (ICD-10-GM) as secondary diagnoses.

We obtained detailed resource use data from the department of management and used them to calculate episode-specific costs according to the general guidelines for unit costing for the German Institute for Hospital Reimbursement [42]. We valued all services a patient received during a stay by their full operating costs to the hospital, i.e. including variable and fixed cost shares. For instance, this included a daily documentation of the intensity of nursing care for each patient and a detailed documentation of all expensive pharmaceuticals and medical devices, which was used to calculate differences in per diem cost. We excluded capital costs, because they are not reimbursed by DRG in the German health care system. We adjusted all costs and reimbursements to € of 2014. Therefore, we used the annually reported average increases in health care costs in German as published by the German Federal Statistical Office [43].

### Analyses

The analyses of costs associated with psychiatric comorbidities were subject to several methodological challenges. First, hospital costs were highly clustered within diagnostic groups. Therefore, we controlled for main-diagnoses, i.e. the retrospectively coded reason for the admission, to prevent comparing patients from entirely different diagnostic and therapeutic regimes.

Second, there were no time-stamps attached to the comorbidity data. This complicated the analyses of costs associated with psychiatric comorbidities, as some conditions might have resulted from resource-intensive diagnostic and therapeutic measures during the hospital episode, thereby blurring the attributability of costs. Therefore, we excluded episodes with psychiatric comorbidities that might have occurred during hospital stays (Table 1).

**Table 1:** Diagnostic groups excluded due to potential time dependency

F05	Delirium, not induced by alcohol and other psychoactive substances
F43	Reaction to severe stress, and adjustment disorders
F54	Psychological and behavioral factors associated with disorders or diseases classified elsewhere
F99	Mental disorder, not otherwise specified

Third, increased length of stay itself might have been associated with increased probability of identification and documentation of preexisting psychiatric comorbidities. This sort of time-related bias may inflate estimates if patients with a longer hospital stay and therefore higher costs are then more likely to have a documented psychiatric comorbidity. As time-stamps for secondary diagnosis were unavailable, directly accounting for the time-dependency was not feasible [44]. However, to get a lower bound of our incremental cost estimates in case time-dependency was an issue, we additionally ran our regression adjusting for length of stay in a sensitivity analysis.

We defined *cases* as episodes that had a non-psychiatric main diagnosis and at least one documented psychiatric secondary diagnosis other than those excluded. *Eligible controls* were patients that had no documented psychiatric secondary diagnosis. We applied stratification by 4-digit ICD10 main-diagnosis code using fixed effects to ensure the comparability of different diagnostic and therapeutic regimes. We excluded cases with a main diagnosis from a group in which no controls were available. Vice versa, we excluded potential controls with a main diagnosis from a group without cases. We additionally controlled for age, sex, intensive care hours and other somatic comorbidities using the Charlson Comorbidity Index (CCI) [45].

We analyzed two primary outcomes: (1) costs per episode and (2) reimbursement per episode. Furthermore, we used costs and reimbursement adjusted for length of stay in a sensitivity analysis. As hospital cost and reimbursement were non-normally distributed and right-skewed we used a generalized-linear model [46] with a log link and a Gamma distribution, which was determined using a modified park test [47].

## Results

The descriptive statistics of cases and eligible controls included in our regression model are given in Table 2. Patients with at least one psychiatric secondary diagnosis had higher mean costs, a longer length of stay and more somatic comorbidities than the eligible controls.

**Table 2:** Descriptive statistics of patients included in the analysis

	All		Cases		Controls	
	Mean/%	Standard deviation	Mean/%	Standard deviation	Mean/%	Standard deviation
Costs (€)	4834	9165	10,463	18,996	4570	8334
LOS (days)	7.42	7.79	14.01	14.96	7.11	7.14
Age (years at admission)	59	18	58	18	59	18
CCI	1.97	2.80	3.34	3.26	1.90	2.76
Intensive Care Hours	20	123	67	248	18	114
Sec. diagn. (number)	7	6	14	9	6	5
Died (%)	2		6		2	
Female (%)	48		44		48	
Male (%)	52		56		52	
Observations (number)	158,702		7107		151,595	

*LOS* Length of stay, *CCI* Charlson comorbidity index, *sec. Diag.*, secondary diagnoses

Regression results are given in Table 3. Patients with a psychiatric comorbidity had 40% higher costs. The average episode with psychiatric comorbidities had 1344 € higher costs than the average episode without psychiatric comorbidities within the same main diagnosis after controlling for other comorbidities, age, and sex. Reimbursements were 28% or 1004 € higher, respectively.

**Table 3:** Effects of psychiatric comorbidities [95% Confidence Intervals]

	(1) Costs	(2) Reimbursements
	exp(β)	exp(β)
Psychiatric comorbidity	1.397^***^	1.277^***^
	[1.365–1.429]	[1.250–1.304]
Marginal effect	1345 €^***^	1004 €^***^
	[1236 € – 1451 €]	[907 € – 1100 €]
CCI	1.057^***^	1.043^***^
	[1.055–1.060]	[1.041–1.045]
Intensive care hours	1.004^***^	1.003^***^
	[1.004–1.004]	[1.003–1.003]
Age	1.001^***^	1.000^**^
	[1.000–1.001]	[1.000–1.000]
Sex	1.007^*^	0.991^**^
	[0.999–1.016]	[0.984–0.998]
Observations	158,702	158,702

Notes: Generalized linear regression models with a Gamma distribution and a log link. Regressions used ICD-fixed effect to allow for within primary diagnosis estimation. Columns give the exponentiated coefficients except for the marginal effects. 95% confidence intervals in brackets. *CCI* Charlson comorbidity index,. ^*^p < 0.1, ^**^ *p* < 0.05, ^***^ *p* < 0.01

As discussed, these results may be inflated due to a time-related bias. Table 4 shows the results of the sensitivity analysis controlling for the fact that psychiatric comorbidity detection might also be a result rather than a cause of length of stay. After controlling for length of stay in total and at the intensive care unit, psychiatric comorbidities were associated with an increase in daily costs and reimbursement of 207 € and 151 €, respectively. As psychiatric comorbidities can also prolong length of stay, this approach most likely underestimates the effects. But taken as a minimum lower bound, we can assume the true values are somewhere between our two estimates.

**Table 4:** Sensitivity Analysis- Controlling for length of stay [95% Confidence Intervals]

	Costs	Reimbursements
	exp(β)	exp(β)
Psychiatric comorbidity	1.066^***^	1.043^***^
	[1.051–1.082]	[1.028–1.059]
*Marginal effect*	207^***^	151^**^
	[158–256]	[95–206]
CCI	1.021^***^	1.019^***^
	[1.020–1.023]	[1.017–1.020]
Length of Stay	1.082^***^	1.061^***^
	[1.081–1.083]	[1.060–1.062]
Intensive care hours	1.001^***^	1.001^***^
	[1.001–1.001]	[1.001–1.001]
Age	0.999^***^	0.999^***^
	[0.999–0.999]	[0.999–0.999]
Sex	0.990^***^	0.979^***^
	[0.985–0.996]	[0.973–0.985]
Observations	158,702	158,702

Notes: Generalized linear regression models with a Gamma distribution and a log link. Regressions used ICD-fixed effect to allow for within primary diagnosis estimation. Columns give the exponentiated coefficients except for the marginal effects. 95% confidence intervals in brackets. *CCI* Charlson comorbidity index

^*^p < 0.1, ^**^ *p* < 0.05, ^***^ *p* < 0.01;

## Discussion

The main aim of this study was to analyse the association between psychiatric comorbidities and hospital costs and revenues per inpatient episode. A further aim was to discuss and address the methodological challenges in the estimation of these outcomes based on retrospective data. Psychiatric comorbidities were associated with a substantially higher increase in costs than increase in reimbursements, indicating an inadequate calibration of the German DRG-system.

### Strength and weakness of the study

To the best of our knowledge, our work is the first to discuss both abovementioned issues of possible bias caused by time-dependency in the context of psychiatric comorbidities retrospectively obtained from routine diagnosis data. By excluding cases with secondary psychiatric diagnoses that could potentially occur as a complication rather than as an independent comorbidity, we avoided blurring the attributability of cost differences. Leaving these cases in the regression model resulted in substantially larger estimates (data not shown).

We also discussed the possibility that psychiatric comorbidity detection might be a result rather than a cause of length of stay and showed results after controlling for length of stay. These figures should be regarded as a lower bound of the real differences, since psychiatric comorbidities are likely to be associated with increased length of stay. The calculation of differences in per diem costs was possible because we obtained comparably detailed resource use data, such as daily acuity scores and consumption of expensive pharmaceutical and other materials.

Furthermore, the retrospective design of the study allowed the inclusion of about 160 thousand episodes that were treated during four consecutive years in statistical analyses. The high number of observations and the relatively long study period should improve the confidence in the robustness of the findings to cross-sectional and longitudinal variations in clinical practice. However, relatively little high quality work exists the validity of administrative diagnosis data for research purposes [48], and studies have found both poor [49] and good validity [50]. Furthermore, research has found substantial differences in proclivity of health care providers for making and documenting diagnoses in routine clinical care [51]. It was not possible to investigate the validity of the analysed diagnosis data. However, the comprehensive employment of specialised staff for documentation and verification of diagnosis coding for reimbursement purposes at the study site should strengthen the confidence in the presented results. Nonetheless, the detection of psychiatric comorbidities was probably less consistent than could have been achieved in a prospective study.

### Results in relation to prior studies

The results of our study can be compared to previous research. Creed et al. [25] prospectively studied the association of depression and anxiety with hospital costs in 263 medical inpatients. They found a statistically non-significant increase of 19% in costs per episode after controlling for gender, social class, social benefit, and severity of physical illness. This cost difference lies between the results presented in Table 3 and the lower bound presented in our sensitivity analyses. However, the study of Creed et al. [25] only investigated medical inpatients. Furthermore, their study was not exclusively dedicated to hospital costs, but included other services, such as primary care und community costs. Hence, they applied fixed unit costs to hospital services, a method which should be less able to detect cost difference.

Egede et al. [27] retrospectively studied the association between comorbid depression and hospital costs in about 21,500 individuals with diabetes. They found that the presence of comorbid disorders was associated with a statistically non-significant increase in inpatient costs per case of 31%. This figure is relatively close to the results presented in Table 3 and substantially higher than our lower bound estimation provided in the sensitivity analyses. However, Egede et al. [27] used hospital charges instead of costs, which entails methodological caveats [52]. Furthermore, Egede et al. [27] did not account for the potential time-related-bias that the likelihood of detecting depression is likely to increase with increased length of stay.

Hochlehnert et al. [29] retrospectively studied the association of psychiatric comorbidity and hospital costs and reimbursement in 940 cardiovascular inpatients. They found that psychiatric comorbidities were associated with a 49% increase in hospital costs per case after adjusting for age and sex. Furthermore, they found a reimbursement-cost difference in patients with psychiatric comorbidities that was 901 € less than the reimbursement-cost difference in controls. Both of these values were higher than the results presented in Table 3. However, Hochlehnert et al. [29] did not account for the potential time-related biases associated with (1) the potential occurrence of psychiatric secondary diagnoses as results of resource intensive treatments, such as delirium, and (2) the higher likelihood of detecting psychiatric comorbidities in patients that stay longer. These biases could have led to an overestimation of the effect on hospital costs.

### Implications and further research

This study has found that the presence of psychiatric comorbidities in somatic inpatients is associated with higher costs per episode and per day. This cost difference is not sufficiently accounted for in the German DRG-system. If differences in hospital costs between patient groups are not adequately controlled for in DRG-systems, i.e. the payment is either too high or too low for many patients, strong incentives are created to game the system [11]. Therefore, we suggest intensifying the inclusion of psychiatric comorbidities in the German DRG system. Future research should investigate the appropriate inclusion of psychiatric comorbidities in other health care systems’ payment schemes. In analysis of the cost of somatic diseases in general, psychiatric comorbidities should be controlled for as a relevant cost-increasing factor. Methodologically, we would like to stress the importance of taking into account the two sources of time-related bias discussed in this paper when conducting retrospective analyses.

## Conclusion

Psychiatric comorbidities are associated with an increase in costs per episode in somatic hospital care. These additional costs are not sufficiently covered by increased payments in the German DRG system. The patient classification system underlying the German DRGs should take psychiatric comorbidities into account in order to avoid perverse incentives for health care providers.

## Abbreviations

CCI: Charlson Comorbidity Index
DRG: Diagnosis-related Groups
ICD-10-GM: International Statistical Classification Of Diseases And Related Health Problems, 10th revision, German Modification
LOS: Length of stay
